# Reorganization and Suppression of Store-Operated Calcium Entry in Podocytes of Type 2 Diabetic Rats

**DOI:** 10.3390/ijms24087259

**Published:** 2023-04-14

**Authors:** Konstantin Gusev, Alexey Shalygin, Dmitrii Kolesnikov, Leonid Shuyskiy, Sofia Makeenok, Lyubov Glushankova, Konstantin Sivak, Kirill Yakovlev, Yana Orshanskaya, Guanghui Wang, Andrey Bakhtyukov, Kira Derkach, Alexander Shpakov, Elena Kaznacheyeva

**Affiliations:** 1 Institute of Cytology, Russian Academy of Sciences, St. Petersburg 194064, Russia; k.o.gusev@gmail.com (K.G.); shalygin.alexey@gmail.com (A.S.);; 2 Smorodintsev Research Institute of Influenza WHO National Influenza Centre of Russia, St. Petersburg 197376, Russia; kvsivak@gmail.com (K.S.);; 3 Department of Pharmacology, College of Pharmaceutic Sciences, Soochow University, Suzhou 215031, China; wanggh@suda.edu.cn; 4 Sechenov Institute of Evolutionary Physiology and Biochemistry, Russian Academy of Sciences, St. Petersburg 194223, Russiaalex_shpakov@list.ru (A.S.)

**Keywords:** store-operated calcium entry (SOCE), type 2 diabetes mellitus, TRPC6 channels, ORAI channels, sodium–calcium exchanger (NCX), diabetic nephropathy

## Abstract

Type 2 diabetes mellitus (DM2) is a widespread metabolic disorder that results in podocyte damage and diabetic nephropathy. Previous studies demonstrated that TRPC6 channels play a pivotal role in podocyte function and their dysregulation is associated with development of different kidney diseases including nephropathy. Here, using single channel patch clamp technique, we demonstrated that non-selective cationic TRPC6 channels are sensitive to the Ca^2+^ store depletion in human podocyte cell line Ab8/13 and in freshly isolated rat glomerular podocytes. Ca^2+^ imaging indicated the involvement of ORAI and sodium–calcium exchanger in Ca^2+^ entry induced upon store depletion. In male rats fed a high-fat diet combined with a low-dose streptozotocin injection, which leads to DM2 development, we observed the reduction of a store-operated Ca^2+^ entry (SOCE) in rat glomerular podocytes. This was accompanied by a reorganization of store-operated Ca^2+^ influx such that TRPC6 channels lost their sensitivity to Ca^2+^ store depletion and ORAI-mediated Ca^2+^ entry was suppressed in TRPC6-independent manner. Altogether our data provide new insights into the mechanism of SOCE organization in podocytes in the norm and in pathology, which should be taken into account when developing pharmacological treatment of the early stages of diabetic nephropathy.

## 1. Introduction

Type 2 diabetes mellitus (DM2) is a widespread metabolic disorder primarily characterized by hyperglycemia resulting from a decrease in insulin sensitivity and altered glucose metabolism. One of the complications of DM2 is diabetic nephropathy (DN). DN is characterized by glomerulosclerosis, thickening of the glomerular basement membrane, mesangial cell expansion, podocyte loss and tubulointerstitial fibrosis. Altogether it leads to progressive albuminuria, reduction in glomerular filtration rate, elevation of arterial blood pressure and renal failure [1,2,3].

Damage of podocytes plays a dramatic role during development of DN [1,4]. The most common pathological finding in podocyte disease is a foot process effacement as a result of disrupted podocyte cytoskeleton or the loss of slit diaphragm integrity [5,6,7]. Rearrangement of the actin cytoskeleton strongly depends on the Ca^2+^ homeostasis. 

Disturbances in Ca^2+^ signaling play a central role in podocyte pathophysiology [8]. One of the key regulators of intracellular Ca^2+^ in glomerular podocytes is considered to be TRPC6 (Transient Receptor Potential Canonical 6) channels [9,10,11,12,13]. TRPC6 channels form a relatively non-selective cation channel with single channel conductance of 15–45 pS and could be activated by diacylglycerol (DAG) upon stimulation of G_q/11_-coupled receptors linked to phospholipase Cβ (PLCβ), particularly angiotensin receptors [14,15,16,17]. The decisive contribution of TRPC6 channels to the pathological Ca^2+^ signaling in the kidney has been demonstrated in many studies, especially in the studies of genetically inherited TRPC6 mutations that cause nephropathy. TRPC6 knockout or downregulation in podocytes was protective in several models of diabetes type 1 [10,18,19]. However, a recent publication has questioned the protective role of TRPC6 KO against DN in Sprague–Dawley rats injected with a high dose of streptozotocin (STZ) used to induce type 1 diabetes mellitus [20]. In contrast, the gain-of-function mutation, overexpression or inhibition of TRPC5, which is also expressed in the kidney, does not lead to the podocyte damage [21,22].

Although the role of TRPC6 channels in podocytes is usually associated with direct Ca^2+^ influx, it should be noted that TRPC channels are not very selective. Therefore, Estacion et al. evaluated the relative permeability for Ca^2+^ versus monovalent cations to be around 1.2 and suggested that Ca^2+^ only slightly permeates TRPC6 channels and acts rather as a TRPC6 blocker, inhibiting predominant Na^+^ influx through TRPC6 channels [15]. Recently, the physiological role of Na^+^ influx through TRPC6 channels was demonstrated in primary neurons [23]. It is not known whether a similar mechanism occurs in podocytes. 

The underlying mechanism is based on another important regulator of Ca^2+^ homeostasis in excitable and non-excitable cells, the sodium–calcium exchanger (NCX). The NCX can move Ca^2+^ into and out of cells depending on the net electrochemical gradient, exchanging 3 Na^+^ for 1 Ca^2+^ ions [24]. It was shown that NCX may contribute to agonist-induced and store-operated Ca^2+^ entry in several cell types [25,26,27]. Previously, a functional interaction between TRPC6 channels and NCX in smooth muscle cells and cortical neurons has been shown, when Na^+^ entry through TRPC6 channels favors Ca^2+^ entry by a reverse mode of NCX [23,28,29,30,31].

The main player of store-operated Ca^2+^ entry (SOCE) pathway in non-excitable cells is ORAI channels which are regulated by STIM1/2 (STromal Interaction Molecule) proteins. Moreover, a number of articles suggest that sensitivity of TRPC channels to the Ca^2+^ store depletion may depend in part on ORAI-mediated Ca^2+^ entry [32,33,34,35]. Several publications demonstrated the ORAI role in podocytes. ORAI1 and STIM1 overexpression results in podocyte injury and increased permeability [36]. The incubation of AB8/13 human podocyte cell line with high glucose increased ORAI1 expression and enhanced SOCE, which resulted in calpain activation, cytoskeleton disruption, nephrin reduction and led to podocyte damage [37]. Recently, by using mice overexpressing ORAI1 and mice with ORAI1 or TRPC6 knockout, Kim J et al. [38] demonstrated that ORAI1 but not TRPC6 is critical for insulin-induced albuminuria caused by calcineurin activation and synaptopodin degradation. In addition, in hyperinsulinemic db/db mouse model, authors revealed transient upregulation of ORAI1 expression at the early hyperinsulinemic stage and upregulation of TRPC6 expression prominent at the later stage of DM2 development. 

Several publications demonstrated that TRPC6 channels are insensitive to Ca^2+^ store depletion [38,39,40,41]. However, some other researchers have shown that TRPC6 channels can interact with ORAI and STIM1/2 proteins and are involved in SOCE in different cell types [23,42,43,44]. In some cases, the dependence of TRPC6 channels on Ca^2+^ store depletion could be attributed to the heteromeric composition of TRPC channels with the formation of channels with different biophysical properties [45].

In this article, we studied the Ca^2+^ entry induced by Ca^2+^ store depletion in human podocyte cell line Ab8/13 and in freshly isolated rat glomerular podocytes. Also, we studied the functioning of SOCE after development of DM2 induced in Wistar rats by high-fat diet combined with a low dose of STZ injection. Such a model is used to induce metabolic impairment, including insulin resistance, hyperglycemia and obesity, being useful to study early stages of DN [46,47,48]. We demonstrated the complex interplay of TRPC6, NCX and ORAI during Ca^2+^ entry induced by store depletion. We found that the main source of SOCE in healthy podocytes is ORAI channels while TRPC6 channels modulate Ca^2+^ entry through Na^+^ influx-induced depolarization, which also promotes NCX function in reverse mode and subsequent Ca^2+^ influx. DM2 leads to the pronounced reorganization of the Ca^2+^ signaling, which results in overall suppressed store-operated Ca^2+^ entry in podocytes and could lead to impaired podocyte function.

## 2. Results

### 2.1. TRPC6 Channels and Store-Operated Ca^2+^ Entry in Human Podocyte Cell Line Ab8/13

To study the store-operated Ca^2+^ entry, in the first place, we decided to perform experiments on the human podocyte line Ab8/13. It is an immortalized cell line which was differentiated for 10–14 days at 37 °C (see Section 4). This cell line is often used to model the pathology in podocytes.

**Figure 1:**
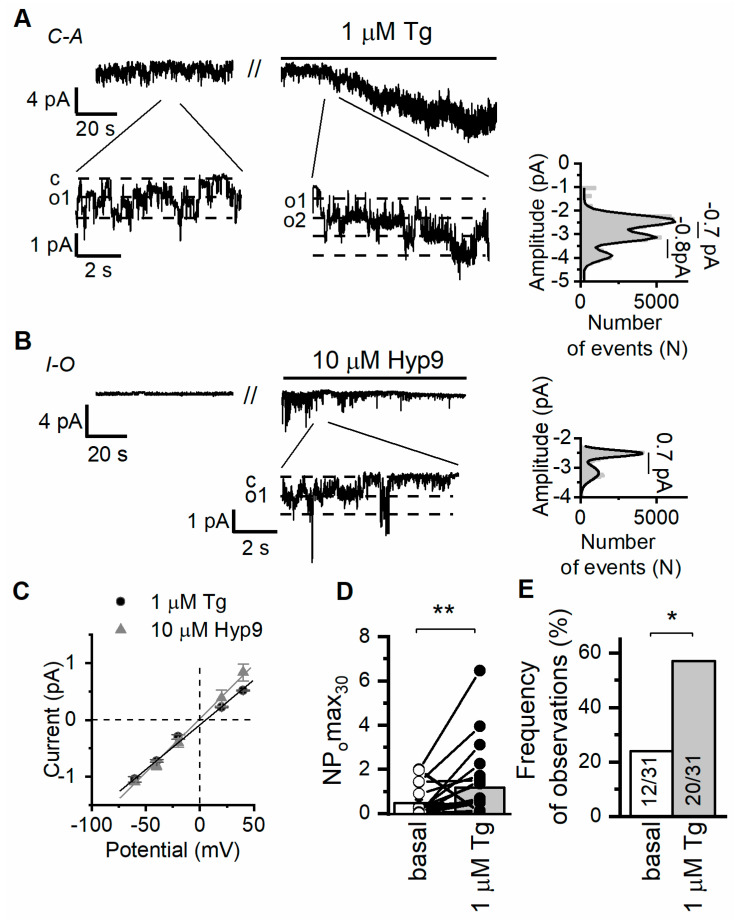
Ca^2+^ store depletion activates TRPC6 channels in human culture podocytes Ab8/13. (**A**) Representative traces of currents recorded in cell-attached configuration in culture podocytes Ab8/13 at holding potential of −40 mV. Shown are basal currents and currents after bath application of 1 µM thapsigargin (Tg). Fragments of the recordings and corresponding amplitude histogram after Tg application are shown at the bottom on an expanded time scale. (**B**) Representative traces of currents recorded in inside-out configurations in Ab8/13 podocytes at holding potential of −40 mV. Shown are basal currents and currents after bath application of 10 µM hyperforin 9 (Hyp9). (**C**) The current–voltage relationship of observed TRPC6 channels in response to Tg and Hyp9. (**D**) The open channel probability collected during 30 s of maximal activity (NPomax30) before and after bath application of 1 µM Tg. Data presented as mean bar plots and paired data points were statistically analyzed using paired sample Wilcoxon signed-rank test. (**E**) The frequency of TRPC6 channel observation as portion of experiments. In bar plot are shown the number of experiments with TRPC6 activity over total number of performed experiments. Data were statistically analyzed by Barnard’s exact test. * and ** indicate *p* values of <0.05 and <0.01, respectively.

In our electrophysiological experiments on Ab8/13 podocytes, we performed cell-attached measurements of single channel currents induced by calcium store depletion with 1 µM thapsigargin (Tg). The ORAI channels have very small amplitude and could not be measured on a single channel level. In the Ab8/13 podocytes, we observed mostly the channels with a conductance of 18 ± 1 pS and a reversal potential close to 0 mV (n = 20; Figure 1A,C). We observed similar channels with conductance of 19 ± 3 pS (n = 3) also in inside-out mode of patch-clamp upon application of 10 µM hyperforin 9 (Figure 1B,C). In contrast to the DAG, hyperforin is known to selectively activate TRPC6 over other TRPC channels [49].

Previously, in CHO cells overexpressing human TRPC6 channels, we recorded single channels with an estimated conductance of 20 ± 1 pS in cell-attached mode [9]. Ilatovskaya et al. also revealed channels with similar characteristics and conductance of 22 pS in freshly isolated glomerular mouse podocytes, which were absent in TRPC6KO mice [16]. Thus, the characteristics of the recorded channels resemble the characteristics of TRPC6 channels (Figure 1). However, we cannot fully exclude that other TRPC isoforms formed a heteromeric channel with TRPC6 and contributed to the recorded single channel currents.

It is important that application of 1 µM Tg increased frequency of observation and activity of TRPC6 channels (Figure 1A,D,E). Therefore, in our experiments, TRPC6 channels are induced downstream to store depletion and can potentially contribute to store-operated Ca^2+^ entry (SOCE) in human podocyte cell line.

**Figure 2:**
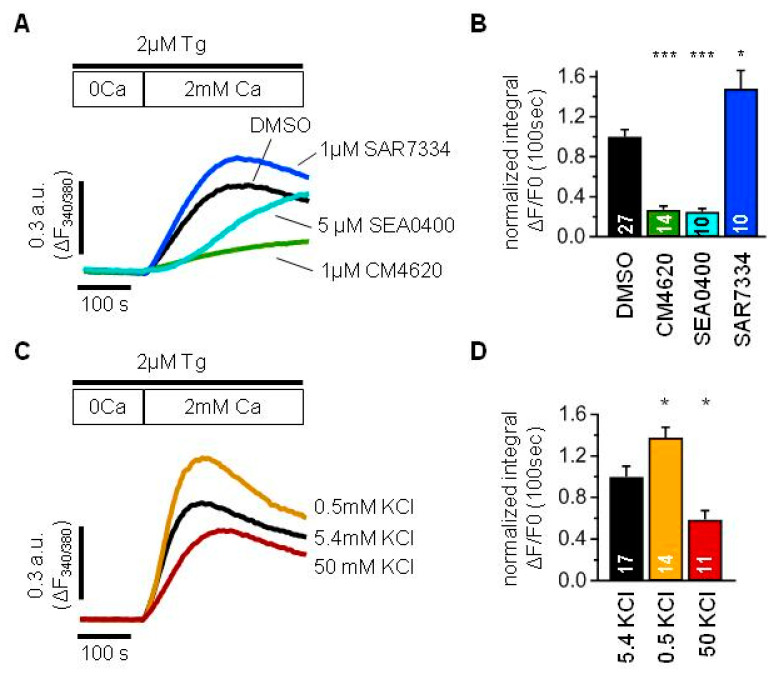
TRPC6 channels modulate store-operated Ca^2+^ entry mediated by ORAI and NCX in human culture podocytes Ab8/13. (**A**,**C**) An averaged traces demonstrating ratio of fura-2 fluorescence at 340 and 380 nm, which reflect changes in intracellular Ca^2+^ concentration in human podocytes Ab8/13. Cells were incubated with 2 µM Tg in 0 mM Ca^2+^ solution for 10 min and afterwards 2 mM Ca^2+^ solution was added to register store-operated Ca^2+^ entry. (**B**,**D**) Bar plots showing the normalized fura-2 fluorescence ratio integrated over 100 s right after addition of 2 mM Ca^2+^. (**A**,**B**) Shown are control Ca^2+^ response (black), Ca^2+^ response in the presence of 1 µM CM4620 (green), Ca^2+^ response in the presence of 1 µM SAR7334 (blue) and Ca^2+^ response in the presence of 5 µM SEA0400 (light blue). (**C**,**D**) Ca^2+^ entry recorded in the presence of 5.4 mM KCl used as control (black), 0.5 mM KCl (gold), 50 mM KCl (dark red). Data were statistically analyzed by Kruskal–Wallis test followed by Dunnett’s test. *, and *** indicate *p* values of <0.05, and <0.001, respectively.

To reveal the contribution of TRPC6 channels to SOCE, we performed Ca^2+^ imaging experiments with fura-2 as Ca^2+^ indicator (Figure 2). In addition, we checked the effect of different inhibitors on the SOCE in podocytes.

Podocytes were kept in normal Tyrode solution before experiments, washed with 0 Ca^2+^ solution and a minute later 2 µM Tg was applied for 10 min together with a corresponding inhibitor or DMSO as a control. Then, 2 mM Ca^2+^ were reconstituted to the extracellular solution and the Ca^2+^ influx was analyzed by integrating over the first 100 s after Ca^2+^ addition (Figure 2A,B). 

The application of 1 µM CM4620, a selective inhibitor of ORAI1 channels, significantly suppressed Tg-induced SOCE (Figure 2A,B). This implies a great involvement of ORAI1 channels into the store-operated Ca^2+^ entry in podocytes. 

Surprisingly, the application of 1 µM SAR7334, a selective inhibitor of TRPC6 channels, in spite of expectations, resulted in increased Ca^2+^ entry (Figure 2A,B). Therefore, it could be suggested that TRPC6 channels play rather a negative role in SOCE. 

One of the explanations could be that TRPC6 channels conduct more Na^+^ ions than Ca^2+^ ions, which will lead to the membrane depolarization and decrease in Ca^2+^ entry due to the reduction in electrochemical gradient for ORAI channels.

To test the effect of membrane polarization on the SOCE in AB8/13 podocytes, we modulated the membrane potential by varying the extracellular K+ concentration. As expected, the SOCE was significantly reduced in the solution containing 50 mM KCl used to depolarize the plasma membrane (Figure 2C,D). In contrast, the SOCE was significantly higher in the solution containing 0.5 mM KCl used to hyperpolarize the plasma membrane (Figure 2C,D).

From another side, Na^+^ influx through TRPC6 channels can lead to the Ca^2+^ entry through NCX working in reverse mode.

To check an involvement of NCX into the SOCE, we applied 5 µM SEA0400, a selective inhibitor of NCX, which resulted in the SOCE suppression (Figure 2A,B). Although it was almost absent at the later phase, the effect was significantly pronounced during the earlier onset of the Ca^2+^ response. Thus, the NCX is also an important determinant of intracellular Ca^2+^ increase in human podocytes and should be taken into account.

Taken together, the store depletion induces intracellular Ca^2+^ increase in Ab8/13 podocytes mostly by ORAI channels with pronounced contribution of NCX. The TRPC6 has rather negative impact on the ORAI-mediated Ca^2+^ entry and positive impact on the NCX-mediated Ca^2+^ entry. In human podocytes Ab8/13, the negative effect of TRPC6 function prevails over its positive effect, which results in SOCE suppression.

### 2.2. Type 2 Diabetes Induction with High-Fat Diet and Low-Dose Streptozotocin

In the Wistar male rats, DM2 was induced by a 16-week high-fat diet (HFD) and treatment with a low-dose STZ 20 mg/kg (after 10 weeks of diet). The rats with DM2 had a significant increase in the body and kidney weights, the increased blood levels of glucose, glycated hemoglobin, total triglycerides and total cholesterol (Table 1). Diabetic animals were characterized by impaired glucose tolerance, as indicated by an increase in the AUC0-120 value by 47% compared with that in control animals, as well as a significant increase in glucose levels 30, 60 and 120 min after a glucose load during IGTT. In diabetic rats, both basal and glucose-stimulated (120 min after glucose loading) levels of insulin and leptin also increased, indicating the development of hyperinsulinemia, hyperleptinemia, and resistance to insulin and leptin, which are characteristic features of DM2 (Table 1). In addition, diabetic rats had an increased level of creatinine, a decreased level of the creatinine clearance, and proteinuria (Table 1). These data indicate not only the development of DM2 signs in animals, such as obesity, impaired glucose tolerance, insulin resistance and dyslipidemia, but also the development of renal dysfunctions.

To assess the glomerular injury, we analyzed Masson Trichrome stained right kidney slices. In spite of the diabetes development and described above changes, glomeruli from DM2 rats had no obvious structural damages. The kidney capsule was without any defects and thickening throughout. Also, the capillary features were without any signs of microcirculatory disorder. Capillary walls were without signs of sclerosis, hyalinosis and plasma soaking.

**Figure 3:**
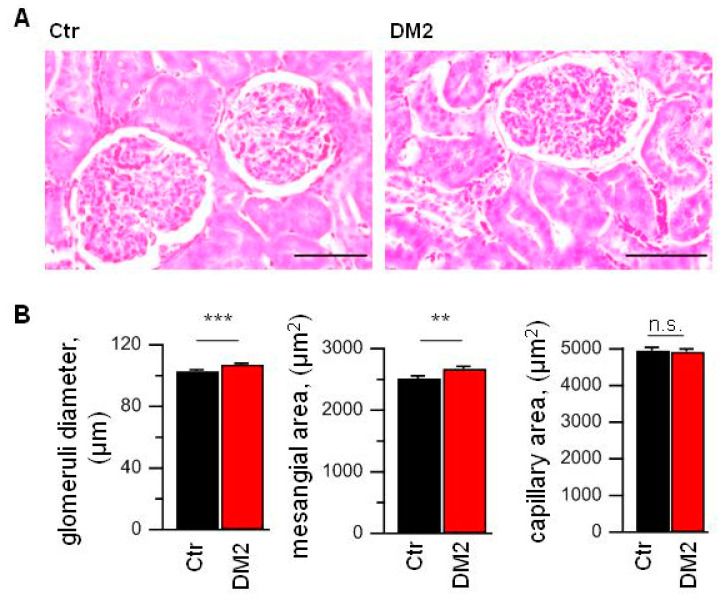
High-fat diet and STZ injection results in the glomerular hypertrophy and mesangial expansion. (**A**) Masson Trichrome stained slices from control (Ctr) and diabetic (DM2) right kidney. Scale bar equal 50 µm. (**B**) The averaged data obtained from analysis of right kidney slices (N = 9 rats per group; n= 450 glomeruli per group). Data were statistically analyzed by Student *t*-test. n.s., not significant, ** and *** indicate *p* values of <0.01, and <0.001, respectively.

To assess the glomerular changes, 50 glomeruli per animal for all animals in control and DM2 groups were picked at random (N = 9 rats per group). The analysis revealed an increase in mesangial matrix expansion and an increase in glomerular diameter, which indicates some hypertrophy (Figure 3). The capillary area was unchanged, so the capillary to mesangial ratio index was reduced.

It should be noted that rats are quite resistant to nephropathy development. Therefore, in order to induce the diabetic nephropathy, investigators use salt-sensitive rats or heminephrectomized rats [1,50]. In current work, we used Wistar rats on a high-fat diet with STZ injection which allows studying early changes leading to diabetic nephropathy.

### 2.3. Decrease of Tg-Induced TRPC6 Single Channel Activity in Podocytes of Type 2 Diabetic Rats

**Figure 4:**
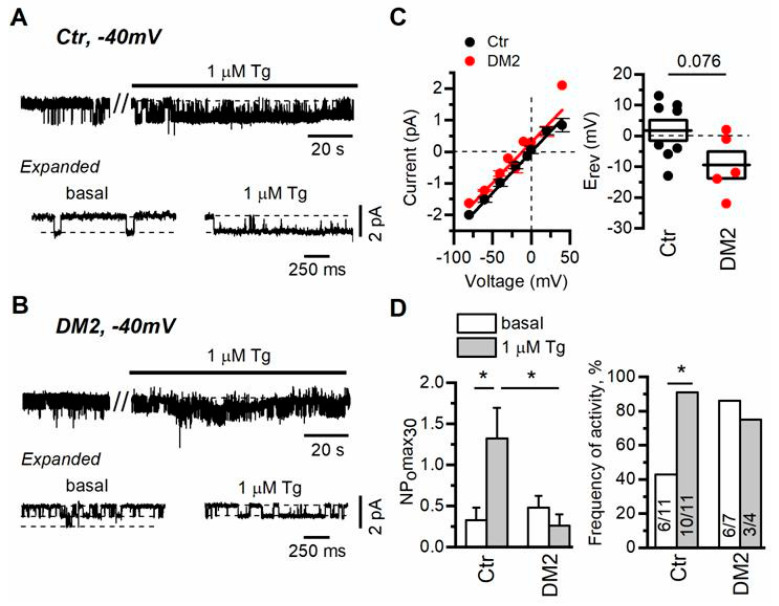
Diabetes development cancels the regulation of TRPC6 channels by Ca^2+^ store depletion in rat podocytes. (**A**,**B**) Representative traces of TRPC6 currents recorded in cell-attached configuration in freshly isolated rat glomerular podocytes from control (**A**) and diabetic (**B**) groups. The membrane potential was held at −40 mV. Shown are basal currents and currents after bath application of 1 µM thapsigargin (Tg). Fragments of the recordings are shown at the bottom on an expanded time scale. (**C**) The current–voltage relationship and reversal potential of observed TRPC6 channels. Data were analyzed by two-tailed Student *t*-test. (**D**) Left—the open channel probability collected during 30 s of maximal activity (NPomax30) before and after bath application of 1 µM Tg. Data are presented as mean bar plots and paired data points. Line indicates the median value. Data were statistically analyzed by Kruskal–Wallis test followed by Dunnett’s test. (**D**) Right—the frequency of TRPC6 channel activity observation as percentage of all patches with TRPC6 activity in the group. In the bars are shown the number of active recordings at these conditions over total number of recordings with activity. Data were statistically analyzed by Barnard’s exact test. * indicate *p* values of <0.05.

To study store-operated channels in rat glomerular podocytes freshly isolated from diabetic and control rats, in the first place, we also used cell-attached voltage-clamp recordings. Application of 1 µM Tg resulted in TRPC6 channel activation with conductance of 24 ± 2 pS (Figure 4A,C). In addition, we observed Tg-induced activation of the channels with conductance of about 6.5 ± 0.4 pS, which we did not analyze further because of rare observation and complication in analysis upon activation together with TRPC6 channels. We, as well as another group, have already registered and described these channels in rat podocytes earlier [9,51].

In our experiments, similarly to the experiments of another group [16], we did not reset the membrane potential to zero with high KCl containing solution, which means that the resting potential of the cell had impact on the observed reversal potential of recorded channels. This approach allowed us to determine a slight shift in the registered reversal potential of TRPC6 channels in diabetic versus control podocytes, which reflects rather the depolarization of plasma membrane in diabetic podocytes (in control podocytes Erev = 1.8 ± 3.3 mV, n = 8; in diabetic podocytes Erev = 9.4 ± 4.4 mV, n = 5, *p* = 0.076) (Figure 4C). The shift in membrane potential also explains slightly different single-channel amplitude observed at −40 mV (Figure 4A,B).

In control podocytes, the TRPC6 channel activity, expressed as NPomax30, was increased from 0.3 ± 0.2 to 1.3 ± 0.4 after 1 µM Tg application (Figure 4D). In podocytes from rats with diabetes, we observed that basal TRPC6 activity was slightly increased (NPomax30 = 0.5 ± 0.1), while the influence of Ca^2+^ store depletion on TRPC6 activity was negligible (NPomax30 = 0.3 ± 0.1, Figure 4B,D). Moreover, in diabetic podocytes, the percentage of recordings with active TRPC6 channels (NPomax30 > 0.05) before and after Tg treatment was unchanged in contrast to the recordings in control podocytes, which may indicate that most of the channels are preactivated in diabetic podocytes (Figure 4D). This may partially explain the shift in the reversal potential of registered TRPC6 channels in DM2 podocytes. Altogether it suggests that in diabetic podocytes, TRPC6 channel activity is practically unaffected by Tg-induced store depletion.

**Figure 5:**
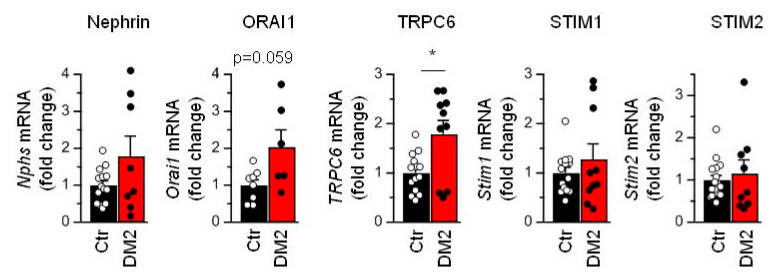
Diabetes leads to the increase in TRPC6 mRNA expression level. Shown are averaged data and individual data points for expression of Nephrin, Orai1, TRPC6, STIM1 and STIM2 mRNA. Data were analyzed by two-tail Mann–Whitney test. * indicate *p* values of <0.05.

Next, we checked the level of nephrin, Orai1, TRPC6, STIM1 and STIM2 mRNA expression. To analyze the expression level, we used glomeruli isolated from rat kidneys by sieving protocols. Real-time PCR revealed significant changes only in the expression level of TRPC6 gene. The TRPC6 mRNA level was increased by 78 ± 28% in glomeruli from diabetic rats (Figure 5), which coincides with data on TRPC6 expression obtained earlier in FGSS nephropathy and in rats with diabetes type 1 [10]. The expression level of ORAI1 was also higher by 103 ± 47%, although the difference did not reach a significant level.

### 2.4. DM2 Developments Result in Reorganization of Store Depletion-Induced Ca^2+^ Entry in Podocytes of Type 2 Diabetic Rats

**Figure 6:**
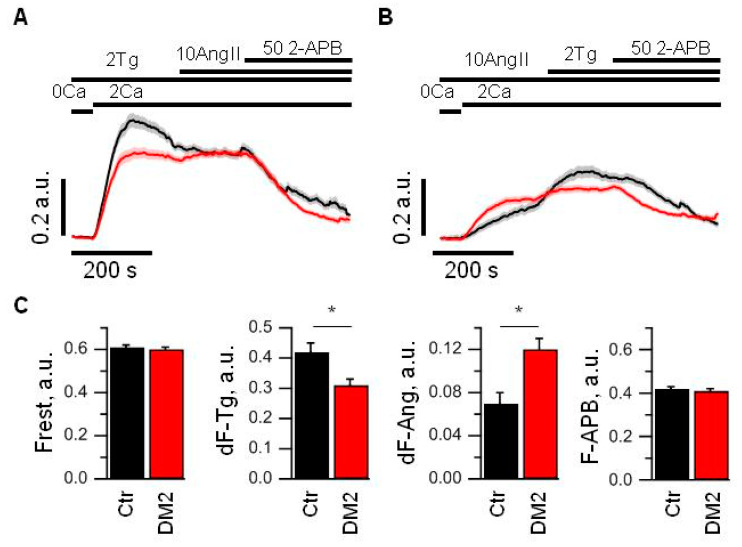
Store-operated Ca^2+^ entry is decreased and receptor-activated Ca^2+^ entry is increased in glomerular podocytes from diabetic rats. (**A**,**B**) An averaged ratio of fura-2 fluorescence at 340 and 380 nm, which reflect changes in intracellular Ca^2+^ concentration in control (black) and diabetic (red) podocytes. Podocytes were stimulated with 2 µM Tg or 10 µM Ang II for 4 min in 0 mM Ca^2+^ solution prior to 2 mM Ca^2+^ addition. The bars above the graph indicate the time when 0 or 2 mM Ca^2+^ was used, when 2 µM Tg, 10 µM Ang II or 50 µM 2-APB were added. (**C**) Bar plots summarizing the analyzed parameters from control (Ctr, black) and diabetic (DM2, red) groups measured from the data shown in panel A and B. Indicated are resting fluorescence ratio (Frest, which reflects the basal level of [Ca^2+^]_i_), an averaged amplitude of Tg-induced Ca^2+^ entry (dF-Tg, measured from experiments shown in panel A), an averaged amplitude of Ang II-induced Ca^2+^ entry (dF-Ang, measured from experiments shown in panel B), and averaged fluorescence obtained after application of 50 µM 2-APB (F-APB, measured from experiments shown in panel A). Data were analyzed by Mann–Whitney test. * indicate *p* values of <0.05.

To study store-operated Ca^2+^ entry in glomerular podocytes freshly isolated from rats, we performed fluorescent imaging of fura-2AM loaded cells. The diabetic and control cells had similar resting levels of Ca^2+^ (Figure 6A,C). To induce store-operated Ca^2+^ entry, glomeruli were incubated in Ca^2+^-free medium and 1 min later 1 µM Tg was applied for 4 min. Then, the extracellular solution was exchanged for the solution with 2 mM Ca^2+^, and we observed Ca^2+^ entry into cytoplasm. The Tg-induced calcium entry in podocytes from diabetic animals was reduced by more than 25% relative to calcium entry in podocytes of control rats (Figure 6A,C). To reveal how the Tg-sensitive pathway intercross with Ca^2+^ entry by activation of G-protein coupled receptor pathway, we applied 10 µM Ang II after 3 min of Tg-induced Ca^2+^ entry. In control podocytes, it resulted in slowing of the Ca^2+^ response rundown, while in diabetic podocytes, it led to the slight increase in Ca^2+^ response (Figure 6A). Moreover, when we performed alternative experiments in which we first activated angiotensin receptors by 10 µM Ang II application, it revealed that Ang II-induced Ca^2+^ entry is increased by more than 50% in diabetic rats as compared to control podocytes, but the subsequent application of 1 µM Tg induced additional Ca^2+^ entry in control and diabetic podocytes (Figure 6B,C). This Tg-induced increase in Ca^2+^ influx amplitude in control podocytes was significantly higher than in diabetic podocytes (Figure 6C).

The application of 50 µM 2-APB, a well-known inhibitor of store-operated Ca^2+^ channels, at the end of the experiments, inhibited both Tg- and Ang II-induced Ca^2+^ influx in control and diabetic podocytes to a similar level. 

All above experiments allow us to conclude that the Ca^2+^ entry caused by Ang II is a part but not the whole of the calcium entry induced by Ca^2+^ store depletion and vice versa. Moreover, Tg- and Ang II-induced pathways of Ca^2+^ entry seem to be regulated in opposite directions upon DM2 development, which suggest some compensatory mechanism. 

In order to further reveal the underlying mechanisms of the impaired Ca^2+^ influx, we performed an inhibitory analysis. In this analysis we looked for the difference in the Ca^2+^ response development in the presence and the absence of a certain inhibitor influencing the Ca^2+^ transport. This allowed us to highlight the contribution of particular components of store-operated Ca^2+^ entry. 

**Figure 7:**
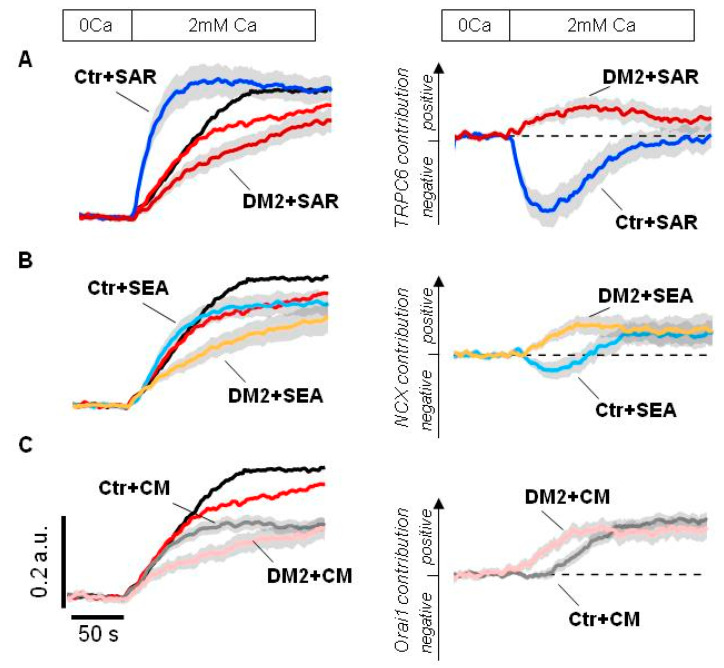
An inhibitory analysis of Ca^2+^ influx into the rat glomerular podocytes. (**A**–**C**) Shown are averaged fura-2 fluorescence ratio in response to Ca^2+^ store depletion by application of 2 µM Tg for 4 min in glomerular podocytes from control (Ctr, black) and diabetic (DM2, red) rats. Left—original traces aligned to the fluorescence ratio level before Ca^2+^ addition; shown are traces recorded in the presence of DMSO or indicated inhibitor. Right—difference between Ca^2+^ response in the presence of DMSO minus Ca^2+^ response in the presence of indicated inhibitor, the ordinate axis reflects the positive and negative contribution of the indicated pathway, estimated by the effect of corresponding inhibitor. Inhibitors were applied in 0 mM Ca^2+^ for 4 min together with 2 µM Tg and kept till the end of experiment. 1 µM SAR7334 (panel A), 5 µM SEA0400 (panel B) and 1 µM CM4620 (panel C) were used. The scale bars shown on the left are the same for all panels. Traces shown as Mean ± S.E.M. values.

First of all, we checked the effect of SAR7334, an inhibitor of TRPC6 channel. In control podocytes, the 1 µM SAR7334 application resulted in an immediate strong potentiation of Tg-induced Ca^2+^ entry, which is similar to the SAR7334 effect in human Ab8/13 culture podocytes (Figure 2A,B and Figure 7A). Interestingly, the application of 1 µM SAR7334 to the diabetic podocytes resulted in a quite different effect—the Ca^2+^ entry was inhibited instead of the potentiating effect observed in control podocytes (Figure 7A). 

The contribution of TRPC6 channels could be more obviously seen by comparative analysis of inhibiting effect of 1 µM SAR7334. As seen in Figure 7A right, TRPC6 involvement in Ca^2+^ signaling is significantly rearranged in podocytes of diabetic animals.

Then, we tested the effect of NCX inhibition by 5 µM SEA0400 during the Ca^2+^ store depletion. In control rat podocytes, the inhibition of NCX resulted initially in moderate Ca^2+^ increase which was followed by decrease in Ca^2+^ entry (Figure 7B). This might reflect bidirectional function of NCX, the removing Ca^2+^ entered through ORAI channels at the initial stage and subsequent contribution to the Ca^2+^ entry, exchanging extracellular Ca^2+^ for cytosolic Na^+^ entered into the cell through TRPC6 channels. In diabetic podocytes, NCX functions in reverse mode and contributes to the Ca^2+^ entry from the very beginning probably due to increased basal TRPC6 activity (Figure 7B right).

Finally, the application of 1 µM CM4620, an inhibitor of ORAI1 channels, had the similar impact on the Ca^2+^ entry in control and diabetic rats, except for some delay in control podocytes (Figure 7C). This delay could probably be explained by a different involvement of NCX and TRPC6 pathways into the Ca^2+^ response, which was described above. 

Altogether, the inhibitory analysis revealed the loss of TRPC6-mediated inhibitory effect on ORAI-dependent Ca^2+^ entry during store depletion, which is in agreement with decreased sensitivity of TRPC6 to the Ca^2+^ store depletion observed in cell-attached experiments (Figure 4). It seems that whereas in control podocytes ORAI is actively suppressed by TRPC6-mediated Na^+^ influx, in diabetic podocytes the inhibition of TRPC6 channels does not recover the ORAI-dependent Ca^2+^ entry (Figure 7A). In addition, in diabetic podocytes NCX starts to function in the reverse mode earlier, providing Ca^2+^ influx.

## 3. Discussion

Diabetes mellitus affects millions of patients worldwide. One of its complications is the development of diabetic kidney disease (DKD). DKD is a result of multiple progressive damages which occur in glomerular cells, tubules and capillaries in the kidney [1,10]. The damage of podocytes leads to disturbances in kidney functioning and development of albuminuria. 

In our study, we used Wistar male rats fed a high-fat diet and injected with a low dose of STZ. Similar protocols for DM2 induction were used previously [50,52,53,54]. In agreement with other studies [47], glomeruli from these rats demonstrate no severe structural disturbances or mesangial matrix accumulation, but have some hypertrophy (Figure 3). The diabetic rats also developed signs of proteinuria and have reduced glomerular filtration rate (Table 1), which probably reflects the early onset of diabetic nephropathy [1].

In our in vivo experiments, we limited ourselves to studying the mechanisms of calcium signaling in the podocytes of diabetic male rats. There is evidence that in diabetes mellitus, kidney damage, including functional changes in podocytes, may be sex-dependent [55,56,57,58,59,60]. This is due to differences in the severity and pathogenesis of diabetes-induced hypertension in men and women, as well as the effect of sex hormones on kidney cells. The degree of kidney damage in men with diabetes is usually higher than in women, as indicated by the results of clinical studies [61,62]. It was also shown that in diabetic male mice, angiotensin-converting enzyme 2 deficiency to a greater extent than in female mice exacerbated angiotensin-II-mediated glomerular hypertrophy, mesangial enlargement, and podocyte loss, and castration of diabetic male mice attenuated kidney damage [59,63]. Despite the fact that in the present study we studied the fundamental cellular mechanisms responsible for calcium signaling in podocytes, we cannot exclude the role of gender factors in the severity of their dysfunctions in diabetes.

Previously, it was shown that in rat glomerular podocytes, TRPC6 channels can be directly activated by Ang II [16]. Ang II leads to PLCβ activation and a DAG generation, a known activator of TRPC6 channels [14,15]. At the same time, PLC activation leads to the formation of inositol 1,4,5-triphosphate, which, in turn, stimulates intracellular Ca^2+^ release and Ca^2+^ store depletion. Previously, it has been shown that in stably transfected mTRPC6 HEK293 cells, the TRPC6 channels are translocated to plasma membrane during either a stimulation of muscarinic receptors or Tg-induced Ca^2+^ store depletion. It was suggested that such externalization does not activate the TRPC6 channels by itself and that another component, such as DAG or ROS, is required for TRPC6 activation [38,64,65,66,67]. Recently, different research groups revealed that TRPC6 channels interact with ORAI1-3 channels and are involved in store-operated Ca^2+^ entry [42,44,45]. Moreover, in breast cancer cells, TRPC6 was shown to be required for the Tg-induced translocation of ORAI1 and ORAI3 channels to the membrane [43]. 

In our previous study, we have demonstrated that disruption of actin cytoskeleton activates TRPC6 channels in both CHO cells and in rat podocytes [9]. In a current study, we observed that application of 1 µM Tg and subsequent Ca^2+^ store depletion increases the activity of TRPC6 channels, as demonstrated by single channel recordings, both in cultured human Ab8/13 podocytes and in isolated rat glomerular podocytes (Figure 1 and Figure 4). The important observation is that in podocytes from diabetic rats, this TRPC6 dependence on Ca^2+^ store filling state is practically lost (Figure 4B,D). In addition, in glomerular podocytes from diabetic rats, we observed a slight shift in a reversal potential of TRPC6 channels (Figure 4C), which indicates the changes in resting potential of podocytes. The shift in a reversal potential of TRPC6 channels could be caused by an increase in channel activity at resting conditions. Similar shift in membrane potential of podocytes was also observed in other studies and suggests a membrane depolarization in podocytes upon TRPC6 activation [67,68]. The higher level of TRPC6 gene expression in glomeruli from diabetic rats suggests an increased number of TRPC6 channels (Figure 5). In Ca^2+^ imaging experiments, we also observed the increase in Ang II-induced Ca^2+^ response in diabetic podocytes (Figure 6B,C). The higher Ang II-mediated Ca^2+^ response accompanied by increase in TRPC6 expression was observed earlier in type 1 diabetes [11]. 

Our Ca^2+^ measurements revealed that in rat glomerular podocytes, store-operated and receptor-operated Ca^2+^ entry pathways partially overlap (Figure 6). Moreover, during the development of diabetes, these pathways appear to undergo significant rearrangement, and SOCE is downregulated while receptor-activated Ca^2+^ entry is upregulated. The increase in the TRPC6 expression and the loss of Ca^2+^ store dependence also coincides with decreased response to Tg and increased response to Ang II observed in podocytes from diabetic rats (Figure 6).

In early studies, the permeability ratio of Ca^2+^:Na^+^ for TRPC6 was estimated to be around 5 [14]. However, later, Estacion et al. evaluated the relative permeability for Ca^2+^ versus monovalent cations to be around 1.2 and suggested that Ca^2+^ only slightly permeates TRPC6 channels and acts rather as a TRPC6 blocker [15]. Moreover, high extracellular Ca^2+^ also decreases the unitary conductance of TRPC6 channels. Thus, at physiological conditions, inward TRPC6 currents are carried mostly by Na^+^, which was also observed upon measurements of single TRPC6 channels [69]. Therefore, the primary effect of TRPC6 activation would be Na^+^ entry and membrane depolarization. We hypothesized that the TRPC6-mediated Na^+^ entry and depolarization may also have an impact on the function of NCX. Overall, these observations complicate the direct conclusions from Ca^2+^ imaging experiments about TRPC6 channel activity and its role in diabetes.

To clarify the rearrangement of the store-operated Ca^2+^ entry under diabetic condition, we applied some key inhibitors of Ca^2+^ transport systems. 

The main store-operated Ca^2+^ entry pathway is assumed to be ORAI channels, regulated by STIM proteins [70]. Kim J et al. [38] demonstrated that in isolated and cultured mouse podocytes, ORAI1/STIM1 is the main component of SOCE and defines also an insulin effect on Ca^2+^ influx and actin reorganization. In our experiments, we confirmed that inhibition of ORAI1 channels by 1 µM CM4620, a concentration which also may lead to the partial ORAI2 inhibition, greatly suppresses SOCE both in human-cultured Ab8/13 podocytes and in rat glomerular podocytes (Figure 2A,B and Figure 7C). 

The acute TRPC6 inhibition by application of 1 µM SAR7334 resulted in a paradoxical increase in SOCE both in human podocytes and in control rat glomerular podocytes (Figure 2A,D and Figure 7A). This observation is consistent with the assumption that depolarization induced by TRPC6-mediated Na^+^ entry inhibits Ca^2+^ entry in podocytes. This coincides with study of Estacion et al. 2006 [15], where by means of perforated whole-cell recordings combined with Ca^2+^ imaging, it was demonstrated that the primary effect of activating TRPC6 is a membrane depolarization while the channels conduct mostly Na^+^ ions with low contribution of Ca^2+^ to whole-cell currents. However, many studies consider Ca^2+^ permeability of TRPC6 channels, not taking them into account as a predominant pathway of Na^+^ influx [10,71,72]. 

The direct effects of depolarization and hyperpolarization on SOCE were clearly observed by varying the extracellular KCl concentration (Figure 2C). We assume that in podocytes, the depolarization affects Ca^2+^ entry mostly by reduction of Ca^2+^ influx through ORAI channels. In control podocytes, the inhibition of TRPC6 by 1 uM SAR7334 and accordingly removing its depolarizing effect results in strong upregulation of Ca^2+^ influx (Figure 7A). In diabetic podocytes, Tg-induced SOCE is suppressed (Figure 6B,C). However, inhibiting of TRPC6 channels by 1uM SAR7334, in contrast to control podocytes, not only relieved Ca^2+^ influx, but further suppressed it (Figure 7A). 

The reduction of SOCE in diabetic podocytes, observed in both the presence and absence of TRPC6 activity, suggests suppression of ORAI-dependent Ca^2+^ entry during store depletion. While the ORAI expression is not downregulated (Figure 5), it means there are functional changes in ORAI activity. Since expression of STIM1/2 can dramatically influence ORAI activity [34], we checked for the expression level of Stim1 and Stim2 genes. However, we did not observe changes in their expression level (Figure 5). 

In diabetic podocytes, we observed a shift in Erev, which assumes the membrane depolarization (Figure 4C). In addition, earlier involvement of NCX in Ca^2+^ inflow (Figure 7B), assuming similar level of intracellular Ca^2+^ at resting condition (Figure 6C), points to the increased Na^+^ concentration in cytoplasm. The Na^+^ accumulation could be the result of enhanced basal TRPC6 activity or a disturbance in Na^+^ removal, particularly by Na^+^/K^+^ ATPase, which is known to interact with TRPC6 channels in kidneys [73]. The depolarization per se may potentially explain the reduction of ORAI-mediated Ca^2+^ entry in the presence of TRPC6 inhibitor in diabetic podocytes. 

In diabetic podocytes, the reduction of SOCE upon the application of 1 µM SAR7334 may reflect inhibition of either Ca^2+^ influx or Na^+^ influx via TRPC6. Reduced Na^+^ influx may attenuate NCX-associated Ca^2+^ influx in a reverse mode [23,24,28,29,31]. Here, by the use of a specific NCX inhibitor SEA0400 (5 µM), we established that in human and rat podocytes, NCX represents a part of Ca^2+^ entry during Ca^2+^ store depletion (Figure 2A,B; Figure 7B). NCX may be passively involved in Ca^2+^ entry downstream to the store depletion, reflecting the accumulation of Na^+^ in cytosol. It seems that in podocytes from control rats, NCX functions bidirectionally—initial removal of entered Ca^2+^ ions and subsequent bringing of Ca^2+^ ions into the cell (Figure 7B, right). In podocytes from diabetic rats, NCX functions in reverse mode already from the beginning of Ca^2+^ entry after Ca^2+^ readdition, which might occur when cells have already a reduced Ca^2+^ influx and increased intracellular Na^+^ concentration. 

It is important to mention that there is a complicated interplay between ORAI and NCX systems. Thus, inhibiting of Ca^2+^ entry through ORAI channels by CM4620 will impact the intracellular Ca^2+^ level, which favors the Ca^2+^ inflow by NCX. Therefore, the true evaluation of ORAI contribution to SOCE using the inhibitor may be masked by NCX-mediated Ca^2+^ influx. From another side, inhibition of NCX by SEA0400 might influence ORAI function due to changes in intracellular Na^+^ level and its impact on membrane voltage, which complicates the correct estimation of NCX contribution.

Altogether, our data indicate that in healthy podocytes, store depletion leads to the activation of Ca^2+^ influx through ORAI channels and Na^+^ influx through TRPC6 channels. The Na^+^ influx results in a plasma membrane depolarization, a great suppressor of ORAI-mediated Ca^2+^ influx. At the same time, an increasing concentration of cytosolic Na^+^ switches NCX from Ca^2+^ removal to Ca^2+^ inflow in the reverse mode. The contribution of ORAI and NCX/TRPC6 Ca^2+^ entry pathways to the total intracellular Ca^2+^ increase might depend on cell conditions and applied stimulus.

The development of diabetes type 2 results in a significant rearrangement of the whole SOCE system. First of all, in diabetic podocytes, TRPC6 channels lost their dependence on the Ca^2+^ store depletion (Figure 4D and Figure 7A). Herewith, ORAI-mediated/dependent Ca^2+^ entry seems to be suppressed in diabetes (Figure 7A). During diabetes development, the expression level of TRPC6 channels and agonist-stimulated Ca^2+^ response is increased (Figure 5, Figure 6 and Figure 7A), which might lead to the basal accumulation of Na^+^ within the cell, cell depolarization and an earlier contribution of Ca^2+^ influx through NCX (Figure 4C and Figure 7A). Thus, the store-operated Ca^2+^ entry in diabetes undergoes a pronounced reorganization and suppression. 

In summary, our data provide new insights into the mechanism of store-operated Ca^2+^ entry organization in healthy podocytes and after DM2 development.

## 4. Materials and Methods

### 4.1. Cell Culture

The conditionally immortalized human podocyte cell line AB8/13 was kindly provided by M. Saleem and has been described previously [9,74]. Podocytes were cultured in the medium which contained RPMI 1640 medium with 10% fetal bovine serum, and insulin–transferrin–selenium at 33 °C. For the differentiation, podocytes were cultured at 37 °C for 10–14 days. The culture medium and serum were purchase from Gibco, Waltham, Massachusetts, USA

### 4.2. Animals and the Type 2 Diabetes Induction

Adult male Wistar rats were housed in cages with a normal light–dark cycle (12 h/12 h, light on at 09 h) and had a free access to food and water. All experiments were carried out under the Bioethics Committee of Sechenov Institute of Evolutionary Physiology and Biochemistry, Russian Acad. Sci., St. Petersburg, Russia (protocol No. 11, 28 November 2019), and according to “Guide for the Care and Use of Laboratory Animals” and to the European Communities Council Directive of 1986 (86/609/EEC) and all efforts were made to minimize animal suffering and to reduce the number of animals used.

To induce DM2, we used a protocol described earlier [54,75]. Shortly, two-month-old rats were randomly divided into two groups, control (Ctr) and diabetic (DM2). Diabetic rats were fed a high-fat diet (HFD), which included a prescribed amount of high-fat mixture (15 g/day/rat) and dry food given ad libitum (on average: 7–12 g/day/rat), while control animals received a standard food, which included dry food given ad libitum (on average: 20–30 g/day/rat). One kilogram of the mixture consisted of 524 g of pork lard, 417 g of curd, 50 g of liver, 5.3 g of L-methionine, 1.85 g of baker’s yeast and 1.85 g of NaCl. Every morning, diabetic rats of Group DM2 were fed the high-fat mixture. Ten weeks after start of the diabetes induction procedure, the DM2 group was treated with freshly prepared STZ (“Sigma-Aldrich”, St. Louis, MO, USA) in 0.1 M sodium citrate buffer, pH 4.5, which was administered intraperitoneally at a dose of 20 mg/kg of body weight. Control animals received citrate buffer instead of STZ. After treatment with STZ, animals of the diabetic group continued to receive a high-fat diet for six weeks. 

Two days before STZ injection, animals with increased body weight and the increased postprandial glucose level were selected for DM2 induction. Two weeks after STZ treatment, rats with obvious signs of DM2 were selected among the animals of the experimental group, including the increased body weight, increased level of glycated hemoglobin (HbA1c, above 6%), as well as the glucose intolerance according to the results of intraperitoneal glucose tolerance test (IGTT). In the IGTT, 120 min after glucose loading, the glucose level in diabetic rats was above 7 mM, and the AUC0-120 values for the curve “glucose concentration, mM—time, minutes” were above 30% as compared to the average AUC0-120 values in the control group. The AUC0-120 value for animals with developed DM2 (n = 18) was 1695 ± 85, while for control rats (n = 18) it was 1075 ± 44 (*p* < 0.0001), indicating an increase in the AUC0-120 value in diabetic animals by an average of 58%. The second IGTT was carried out 10 days before the end of the trial. Along with the measurement of glucose levels, in some animals from the control and diabetic groups (8 from each group), blood samples were also taken to determine the levels of insulin (before and 30 and 120 min after the glucose loading) and leptin (before and after 120 min after glucose loading). In these animals, the levels of glycated hemoglobin and lipids (total cholesterol, triglycerides) were also assessed.

One week before the end of the trial, the animals from control (Ctr, n = 18) and diabetic (DM2, n = 18) groups were placed into metabolic cages and the urea samples were collected for further analysis. Determination of the concentration of creatinine and protein in the urine of rats was carried out in urine samples collected within 24 h. Determination of creatinine in urine was carried out using the kit “Creatinine-D-Olvex” (“Olvex Diagnosticum”, Saint Petersburg, Russia) based on the Jaffe reaction with deproteinization. Determination of the protein content in the urine was carried out using the kit “Total protein-2-Olvex” (“Olvex Diagnosticum”, Russia) by the colorimetric method using bromophenol blue. Along with this, blood samples were taken from the tail vein, in which the level of creatinine was determined using the kit “Creatinine-D-Olvex” (“Olvex Diagnosticum”, Russia). Based on these data, the creatinine clearance index was calculated using the formula: Cc = (Ccu/Ccb) × Dm, where Cc is the creatinine clearance, Ccu (μmol/L) is the concentration of creatinine in daily urine, Ccb (μmol/l) is the concentration of creatinine in the blood and Dm (μL/min) is minute diuresis. At the end of the trial, the control and diabetic animals were anesthetized and decapitated. Kidneys were isolated and used for further experiments.

### 4.3. The Determination of Blood Levels of Glucose, HbA1c, Insulin, Leptin, Total Cholesterol and Triglycerides

Glucose levels in the blood obtained from the tail vein were measured using the test-strips “One Touch Ultra” (USA) with a glucometer (“Life Scan Johnson & Johnson”, Denmark). The content of HbA1c was measured using the “Multi Test HbA1c System kit” (“Polymer Technology Systems, Inc.”, Whitestown, IN, USA). The levels of insulin and leptin in the rat serum were measured using the ELISA kits “Rat Insulin ELISA” (“Mercodia AB”, Uppsala, Sweden) and “ELISA for Leptin, Rat” (“Cloud-Clone Corp.”, Houston, TX, USA). The concentration of total cholesterol and triglycerides in the blood was measured using the express test MulticareIN “Biochemical Systems International S.p.A.” (Arezzo, Italy).

### 4.4. Intraperitoneal Glucose Tolerance Test

In the control and diabetes groups, the intraperitoneal glucose tolerance test (IGTT) was performed using a single injection of glucose (2 g/kg, i.p.) after 12 h of fasting, as described earlier [54]. The blood samples for measuring glucose, insulin and leptin levels were collected from the tail vein of rats under local anesthesia (Lidocaine, 2%, 2–4 mg/kg b.w.) before (0 min) and 15, 30, 60 and 120 min after glucose load. The IGTT was performed two days before the STZ treatment (for the selection of animals with developed DM) and one week before the end of diabetes induction procedure.

### 4.5. The Rat Glomeruli Isolation

The glomerular isolation protocol has been described previously [76]. Briefly, in anesthetized rats, the abdominal cavity was cut, and the kidneys were excised and placed in a cold RPMI medium containing 5% BSA. All actions were carried out in accordance with the rules of the local ethical committee. The renal capsule was removed, and the kidney cortex was isolated. The cortex of the kidney was cut into small pieces and passed through mesh grid filters with numbers of 80, 120 (sizes of 177 and 125 µm), and collected with mesh filter 200 (74 µm). Collected glomeruli were sediment, and solution was exchanged for the Tyrode’s solution. Most of the glomeruli lost Bowman’s capsule and were suitable for experiments.

### 4.6. Real-Time Quantitative PCR

Total RNA was extracted from the isolated glomeruli samples using the “ExtractRNA Reagent” (Evrogen, Moscow, Russia) according to the manufacturer’s protocol. The samples containing 1 μg of RNA were transcribed to cDNA using the random oligodeoxynucleotide primers and the “MMLV RT kit” (Evrogen, Moscow, Russia). PCR amplification was performed using the mixture containing 10 ng of reverse-transcribed product, 0.4 μM of the forward and reverse primers, and the “qPCRmix-HS SYBR + LowROX kit” (Evrogen, Moscow, Russia). The amplified signals were detected using the “Applied Biosystems^®^ 7500 Real-Time PCR System” (Life Technologies, Thermo Fisher Scientific Inc., Waltham, Massachusetts, USA). The following RT-PCR amplification protocol was used: (I) initial denaturation at 95 °C for 5 min; (II) three-segment amplification and quantification program consisting of 38 cycles: 95 °C for 30 s, 55–56 °C for 10 s, and 72 °C for 30 s; and (III) the ABI Melt Curve program to check for the presence of a single peak and the absence of primer-dimer formation in each reaction containing a template. The primers that were used in the work are presented in Table 2. The expression of the gene encoding 18S rRNA was used as an endogenous control. The relative expression levels of the target genes were calculated by ΔΔC_t_ method.

### 4.7. Histological Analysis

For the histological analysis, we used 18 rats (9 rats per group). After the kidney isolation, the tissue was fixed in 10% neutral formalin (pH = 7.4) for 48 h. Then, the histological material was organized into cassettes, and the tissue samples were subjected to a Histo-Tek VP1 tissue processor (Sakura, Japan) followed by a forming of paraffin blocks. A total of 3 μm sections were stained with Masson’s trichrome and picrosirius. The microscopy was performed on a LEICA DM1000 light microscope. Morphometry and photofixation were carried out using the ADF Image Capture 4.17 software package. Morphometric assessment of kidney tissue included measuring the diameter, area, volume of the renal glomeruli, the area occupied by the mesangial matrix, counted the number of mesangial cells, the area of the microcapillary bed, the ratio of the areas of the capillary bed to the mesangial matrix and assessed diabetic nephropathy. Measurements were performed in 50 randomly selected cortical glomeruli at ×400 magnification in unpolarized light, on sections stained with trichrome. Additionally, at ×400 magnification, the degree of collagen fiber distribution was assessed in polarized light on sections stained with picrosirius. 

### 4.8. Electrophysiology

The cell-attached currents in podocytes from isolated glomeruli were measured with an Axopatch 200B amplifier (Axon Instruments) operated with PClamp software (Axon Instruments, Union City, CA, USA). The recordings were digitized at 5 kHz and filtered at 500 Hz. NPo was determined by using the following equation: NPopen = <I>/i, where <I> and i are the mean channel current and unitary current amplitude, respectively. <I> was estimated from the time integral of the current above the baseline, and i was determined from current records and all-point amplitude histograms. Data were collected from current records after channel activity reached a steady state. Because the channel activity was transient and displayed significant fluctuations, we used NPo collected during 30 s of maximal activity (NPomax30) as a standard way to compare open-channel probability among different experiments. Average NPomax30 values of channel activity from several experiments are presented in the text and on the figures as mean ± SEM. The pipette solution contained (in mM): 126 NaCl, 1.5 CaCl_2_, 10 HEPES, 10 glucose (pH 7.4). To block Cl- and K^+^ channels, we also applied 100 µM niflumic acid, 10 mM TEA-Cl, 10 nM iberiotoxin, 10 µM 4-AP. The bath solution for experiments with Ab8/13 cells contained (in mM): 130 KCl, 1 CaCl_2_, 10 HEPES, 2 MgCl_2_, 10 glucose (pH 7.4). In experiments with isolated glomeruli, 130 KCl were replaced with 130 NaCl in the bath solution. The solutions upon drug application were exchanged with complete bath solution replacement within 1–3 s. All experiments were performed at room temperature.

### 4.9. Ca^2+^ Imaging

Human podocytes AB8/13 grown on glass coverslips and glomeruli attached to poly-L-lysine covered coverslips were loaded with 2 μM Fura-2AM in the presence of 0.025% Pluronic for 40 min at room temperature. The extracellular solution contained (in mM): 140 NaCl, 5.4 KCl, 1 MgCl_2_, 2 CaCl_2_, 0.33 Na_2_HPO_4_, 10 Glucose, 10 HEPES-NaOH (pH 7.4). In experiments with 0.5 and 50 mM KCl, the concentration of NaCl was appropriately adjusted.

Cells were excited at 340 and 380 nm wavelengths for 100 ms every 2 s by using Lambda 10-B filter changer (Sutter Instruments, Novato, CA, USA). Emission fluorescence was registered at >510 nm with a Zyla 4.2 sCMOS camera operated by µManager open-source microscopy software. Further analysis was performed in FiJi/ImageJ software. The change in cytosolic Ca^2+^ concentration was estimated from the ratio of emission fluorescence intensities at 340 and 380 nm excitation wavelengths. The reagents were purchased from Sigma-Aldrich (St. Louis, MO, USA). Fura-2AM was purchased from Thermo Fisher Scientific Inc. (Waltham, MA, USA).

### 4.10. Data Analysis

Data were analyzed using Igor Pro (WaveMetrics, Portland, OR, USA), Origin (OriginLab, Northampton, MA, USA), ImageJ/FiJi, Excel (Microsoft) software.

Statistical comparison was performed using either Student T-test (for normally distributed data) or Wilcoxon–Mann–Whitney U test and Barnard’s exact test (for categorical data). The data are presented as mean value and the standard error of mean (mean ± SEM). The critical level of significance is taken equal to 0.05. Throughout the manuscript *, **, and *** indicate *p* values of <0.05, <0.01, and <0.001, respectively.

## Figures and Tables

**Table 1:** Body, fat and kidney weight, daily water consumption, daily volume of urine, creatinine clearance and the biochemical parameters in rats with type 2 diabetes mellitus induced by 16-week high-fat diet and single low-dose STZ, as compared to control animals. The data are presented as the Mean ± SEM.

Parameters	Ctr	DM2	*p*
Body Weight, g	354.3 ± 8.8 (n = 18)	416.0 ± 6.6 (n = 18)	<0.001
Kidneys weight, g	2.2 ± 0.2 (n = 18)	2.8 ± 0.1 (n = 18)	<0.01
Glucose (fasting), mM	5.21 ± 0.14 (n = 18)	6.18 ± 0.23 (n = 18)	<0.001
Glucose (120 min after glucose load in IGTT), mM	6.13 ± 0.28 (n = 18)	8.61 ± 0.39 (n = 18)	<0.001
AUC0-120, rel. units	1207 ± 44 (n = 18)	1779 ± 71 (n = 18)	<0.001
Insulin (basal), ng/mL	0.70 ± 0.07 (n = 8)	1.13 ± 0.13 (n = 8)	<0.01
Insulin (30 min after glucose load in IGTT), ng/mL	2.38 ± 0.15 (n = 8)	3.28 ± 0.51 (n = 8)	>0.05
Insulin (120 min after glucose load in IGTT), ng/mL	1.22 ± 0.14 (n = 8)	2.31 ± 0.19 (n = 8)	<0.001
Index IR (basal), rel. units	3.53 ± 0.46 (n = 8)	7.61 ± 0.88 (n = 8)	<0.005
Index IR (120 min after load in IGTT), rel. units	6.38 ± 0.95 (n = 8)	20.76 ± 1.58 (n = 8)	<0.001
Leptin (basal), ng/mL	3.35 ± 0.26 (n = 8)	5.48 ± 0.60 (n = 8)	<0.01
Leptin (120 min after glucose load in IGTT), ng/mL	4.50 ± 0.55 (n = 8)	9.52 ± 0.82 (n = 8)	<0.001
HbA1c, %	4.00 ± 0.08 (n = 8)	5.20 ± 0.10 (n = 8)	<0.001
Total triglycerides	1.04 ± 0.04 (n = 8)	1.28 ± 0.09 (n = 8)	<0.05
Total Cholesterol	4.11 ± 0.10 (n = 8)	5.18 ± 0.34 (n = 8)	<0.05
Water consumption, mL/day	33.9 ± 1.7 (n = 18)	23.7 ± 1.9 (n = 18)	<0.05
Urine volume, mL/day	10.8 ± 1.2 (n = 18)	6.4 ± 1.4 (n = 18)	<0.001
Creatinine, blood, μmol/L	68.9 ± 2.1 (n = 18)	100.5 ± 3.8 (n = 18)	<0.001
Creatinine, urea, μmol/L	12.6 ± 1.0 (n = 18)	25.2 ± 2.1 (n = 18)	<0.001
Creatinine clearance, mL/min	1312 ± 56 (n = 18)	769 ± 43 (n = 18)	<0.001
Urea protein, mg/mL	0.39 ± 0.03 (n = 18)	0.69 ± 0.06 (n = 18)	<0.01

**Table 2:** The primers for the study of target and reference genes using real-time PCR.

Genes	Forward/Reverse Sequence	Product Size (bp)	Annealing Temperature (°C)	Genbank
*TRPC6*	(For) TACTGGTGTGCTCCTTGCAG	141	55	NM_053559.1
	(Rev) GAGCTTGGTGCCTTCAAATC			
*Nphs1*	(For) AAGTACGAATGGACCCCTATGAC	176	56	XM_008759187.2
	(Rev) CAGGGCTGTAGGAAACGGGTG			
*Stim1*	(For) ACTCTCCGGAAGCAGCTAGA	124	55	NM_001108496.2
	(Rev) CCTTCGACAACCGAAGGTCA			
*Stim2*	(For) TGGGGATTCAGGCACTTGTT	126	55	XM_008770187.2
	(Rev) CAAAAGTCAGCACAGTCCCAC			
*Orai1*	(For) TGATGAGCCTCAACGAGCAC	234	56	NM_001013982.1
	(Rev) TGATCATGAGGGCGAACAGG			
*18S*	(For) GGACACGGACAGGATTGACA	50	56	NR_046237
	(Rev) ACCCACGGAATCGAGAAAGA			

## Data Availability

The original contributions presented in the study are included in the article; further inquiries can be directed to the corresponding author.
